# New potential eukaryotic substrates of the mycobacterial protein tyrosine phosphatase PtpA: hints of a bacterial modulation of macrophage bioenergetics state

**DOI:** 10.1038/srep08819

**Published:** 2015-03-06

**Authors:** Mariana Margenat, Anne-Marie Labandera, Magdalena Gil, Federico Carrion, Marcela Purificação, Guilherme Razzera, María Magdalena Portela, Gonzalo Obal, Hernán Terenzi, Otto Pritsch, Rosario Durán, Ana María Ferreira, Andrea Villarino

**Affiliations:** 1 Sección Bioquímica y Biología Molecular, Facultad de Ciencias, Universidad de la República, Iguá 4225, CP 11400, Montevideo, Uruguay; 2 Unidad de Bioquímica y Proteómica Analíticas, Institut Pasteur de Montevideo, CP 11400, Uruguay; 3 Instituto de Investigaciones Biológicas Clemente Estable, CP 11600, Montevideo, Uruguay; 4 Departamento de Bioquímica, Universidade Federal de Santa Catarina, CEP 88040-900, Florianópolis, Brazil; 5 Unidad de Biofísica de Proteínas, Institut Pasteur de Montevideo, CP 11400, Montevideo, Uruguay; 6 Cátedra de Inmunología, Facultad de Ciencias-Facultad de Química, Universidad de la República, CP 11600, Montevideo, Uruguay

## Abstract

The bacterial protein tyrosine phosphatase PtpA is a key virulence factor released by *Mycobacterium tuberculosis* in the cytosol of infected macrophages. So far only two unrelated macrophage components (VPS33B, GSK3α) have been identified as PtpA substrates. As tyrosine phosphatases are capable of using multiple substrates, we developed an improved methodology to pull down novel PtpA substrates from an enriched P-Y macrophage extract using the mutant PtpA D126A. This methodology reduced non-specific protein interactions allowing the identification of four novel putative PtpA substrates by MALDI-TOF-MS and nano LC-MS: three mitochondrial proteins - the trifunctional enzyme (TFP), the ATP synthase, and the sulfide quinone oxidoreductase - and the cytosolic 6-phosphofructokinase. All these proteins play a relevant role in cell energy metabolism. Using surface plasmon resonance, PtpA was found to bind immunopurified human TFP through its catalytic site since TFP-PtpA association was inhibited by a specific phosphatase inhibitor. Moreover, PtpA wt was capable of dephosphorylating immunopurified human TFP *in vitro* supporting that TFP may be a bona fide PtpA susbtrate. Overall, these results suggest a novel scenario where PtpA-mediated dephosphorylation may affect pathways involved in cell energy metabolism, particularly the beta oxidation of fatty acids through modulation of TFP activity and/or cell distribution.

M*ycobacterium tuberculosis* (*Mtb*) is the causal agent of tuberculosis (TB) an infectious disease responsible for over 1.7 million human deaths every year (www.who.int). The incidence of new cases of TB has increased mainly due to the emergence of multi-drug resistant strains and the co-infection with HIV^1^. An important pre-requisite for the rapid development of new clinically relevant drugs is the understanding, at the molecular level, of host-bacteria interactions responsible for pathogenesis. *Mtb* is capable of subverting the host immune response, surviving and replicating within host macrophages^2^. However, the discovery of cytosolic mycobacteria challenged the paradigm that *Mtb* exclusively localizes within the phagosome of host cells^3^. Moreover, *Mtb* cytosolic translocation, mediated by the early secreted antigenic target 6kDa (ESAT-6) and its secretion system called ESX-1, correlates with pathogenicity^3^.These observations suggest that *Mtb* targets and modulates the activity of macrophage cytoplasmic components involved in cell signaling pathways associated with vital cellular processes, including inflammatory, metabolic and survival responses. Among other bacterial factors, *Mtb* protein tyrosine phosphatases (PTPs) may be implicated in these modulatory effects and are considered potential drug targets for anti-tuberculosis therapy^4^.

*Mtb* has two PTPs, PtpA and PtpB, which are delivered into the macrophage during infection acting as key virulence factors^5^,^6^,^7^,^8^. PtpA and PtpB lack protein export signal sequences but both have been detected in the culture filtrates of *Mtb* grown *in vitro*^9^,^10^. Recent studies suggested that the SecA2 and ESX/type VII export systems are possible candidates responsible for PtpA export^4^,^11^. In addition, PtpA has been detected into the infected host macrophage cytoplasm by immuno-electron microscopy and Western blot analysis of the cytosolic fractions, and by the expression of neutralizing single-chain anti-PtpA antibodies^7^. The *Mtb* *PtpA*-deletion mutant strain showed reduced survival of the mycobacteria in infected human THP-1 derived macrophages, and expression of PtpA-neutralizing antibodies and inhibitors simulated this effect^7^,^8^. Recently, Chauhan *et al*. generated an *Mtb* mutant (*MtbΔmms*) by disrupting three virulence genes encoding PtpA, PtpB and the acid phosphatase, SapM^12^. This mutant displayed a significantly reduced ability to infect and grow inside human THP-1 macrophages. Moreover, no bacilli were recovered in spleens and lungs of guinea pigs 10 weeks following inoculation with *Mtb*Δmms, suggesting an important role of these phosphatases in the colonization of these organs^12^.

PtpA is a member of the low-molecular-weight PTP (LMW-PTP) family, which does not require metal ions for catalysis^13^. PtpA shows 37% of sequence identity and high structural similarity to its human orthologue HCPTPB. Surprisingly, the human genome encodes for 107 PTPs but only one belongs to the PtpA family which originates four protein isoforms by alternative splicing of a single transcript^14^. Two eukaryotic cytoplasmic proteins were reported as *Mtb* PtpA substrates: VPS33B (Vacuolar Protein Sorting 33B) which is part of the protein complex C required for membrane trafficking and fusion^7^, and the GSK3α (Glycogen Synthase Kinase 3, alfa subunit)^15^. Dephosphorylation of these macrophage components would act as a bacterial mechanism to adapt to macrophage defense response^7^,^16^. On one hand, dephophorylation of VPS33B by PtpA seems to exclude host vacuolar-H^+^-ATPase leading to inhibition of phagosome acidification and maturation^7^,^16^. Secondly, GSK3α dephosphorylation by PtpA would promote an anti-apoptotic pathway, favoring pathogen survival within host macrophage. As tyrosine phosphatases are capable of utilizing multiple protein substrates, thereby providing versatility in phospho-relay signaling networks, the search for specific phosphatase targets is still open and presents an experimental challenge.

The most commonly used biochemical tool for identifying potential PTP substrates is based on the generation of phosphatase mutants (substrate trapping mutants) that retain the ability to bind substrates but are either unable or severely impaired in carrying out substrate dephosphorylation, allowing the isolation of the PTP-substrate complex^17^,^18^,^19^. One of the most common mutants is produced by the substitution of the conserved catalytic aspartate, which assists the E-P formation and hydrolysis, by an alanine residue (D/A mutant). In the *Mtb* PtpA, the conserved catalytic aspartate is the Asp 126, which is not considered crucial in defining substrate specificity^20^. As reported in a kinetic study, the *Mtb* PtpA D126A mutant is characterized by a reduced activity (lower k_cat_) compared to the wt, without substantial K_m_ modification^21^, as also observed for the corresponding mutants of other PTPs^17^,^22^. This methodology has been successfully used in the identification of substrates of eukaryotic PTPs^18^,^19^ but only a few substrates of bacterial PTPs^7^,^23^. The success of this methodology depends on the use of strict conditions during association and washes steps in order to avoid capturing unspecific and abundant proteins. Furthermore, it is often assumed that substrate-trapping mutants retain the structural and substrate binding properties of wt PTPs. However, significant differences may occur, leading to erroneous interpretation and thus invalidating the strategy^24^. Thus, validation of candidate substrates identified using substrate trapping is indispensable.

In this work, we attempted to improve the substrate trapping methodology to obtain novel *Mtb* PtpA substrates. For this aim, we firstly verified the structural and biochemical properties of the PtpA D126A mutant to ensure its adequacy for substrate trapping. Then, we prepared an extract of human macrophage-like THP-1 cells preserving phospho-tyrosine (P-Y) modifications and studied by SPR how PtpA interacted with potential substrates present in this extract. This allowed us to choose stringent experimental conditions to apply during substrate trapping steps to reduce non-specific interactions. Using this improved methodology, we successfully isolated and identified four new putative eukaryotic *Mtb* PtpA substrates. Three are proteins synthesized in the cytosol and then translocated to the mitochondria: (i) the alpha subunit (ECHA) of the trifunctional enzyme (TFP), an essential enzyme of the fatty acid beta oxidation pathway; (ii) the ATP synthase alpha subunit (ATPA); (iii) the sulfide quinone oxidoreductase (SQRD). The fourth protein is the 6-phosphofructokinase (K6PP) a key regulatory enzyme of the glycolysis which localizes in the cytoplasm. We advanced in the validation of one of these candidates, showing *in vitro* that the TFP is a bona fide substrate of PtpA.

## Results

### Structural and functional properties of PtpA D126A substrate trapping mutant

**Figure 1:**
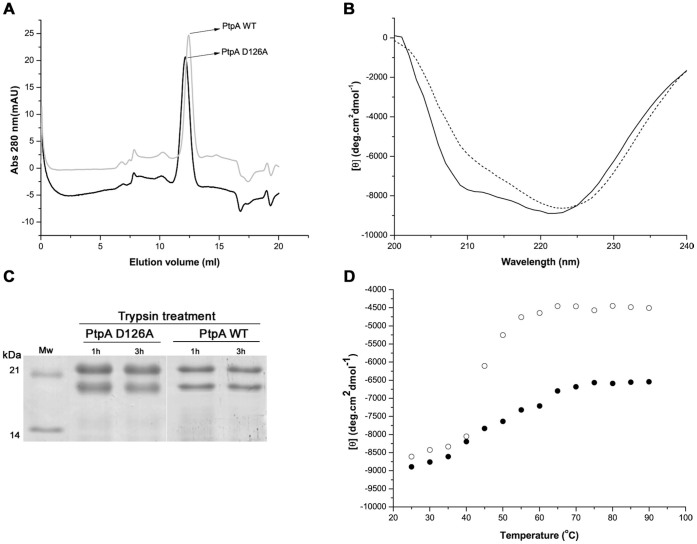
Structural characterization of PtpA D126A. (A) Analysis by size exclusion chromatography of the affinity purified PtpA D126A. The analytical Superdex 75 10/300 GL column was calibrated (y = 4154.9 e^−3.2757×^) using SEC molecular weight markers (SIGMA).The elution volume (Ve) of native PtpA D126A and PtpA wt was 12.16 ml and 12.43 ml, corresponding to 21 kDa and 19 kDa, respectively (theoretical Mw 19.9 kDa). (B) CD spectra of PtpA wt (continuous line) in comparison with that of the mutant D126A (dashed line). These spectra represent the average of five scans performed with PtpA wt and PtpA D126A. (C) SDS-PAGE (15% gel) analysis of the trypsin-treated PtpA D126A and PtpA wt, showing the non cleaved form (~ 20 kDa) and the cleaved form of about 18 kDa; Mw, molecular weight marker. (D) Thermal denaturation curves for PtpA wt (•) and mutant PtpA D126A (o).

To achieve an adequate substrate trapping tool, the mutation introduced into PtpA should not generate substantial structural changes that could impair its functionality. Thus, in this study we generated the PtpA D126A mutant by site-directed mutagenesis and determined its structural and functional properties to assess its suitability for substrate trapping assays. We found that PtpA D126A behaved similarly to the PtpA wt through the whole expression and purification procedure, suggesting that this mutation did not significantly alter the enzyme structure (Supplementary Fig. 1). As found for the wt enzyme, the bulk of PtpA D126A was detected in the soluble fraction following overnight induction at 15°C. Interestingly, the yield of the purified protein, estimated from three biological replicates, was higher for the mutant than for the PtpA wt (8.8 ± 3.13 and 4.6 ± 0.24 mg of protein per liter of bacterial culture, respectively). This difference may be associated with their respective different level in phosphatase activity (see below). In fact, expressing a full active PTP in an organism that already possesses a tyrosine kinase/phosphatase system, as *E. coli*^25^, may cause a cell phosphorylation unbalance and affect the production of the recombinant protein. With regard to the molecular mass, PtpA D126A and PtpA wt were studied by analytical SEC to determine their physical state in aqueous solution. Both phosphatases were eluted in a single major peak with an apparent molecular weight corresponding to the monomeric state (Fig. 1A).

In addition, circular dichroism studies (CD) were undertaken, to assess if the introduced mutation altered PtpA D126A secondary structure^26^,^27^. As shown in Figure 1B, the CD profiles of the wt and PtpA D126A mutant are basically equal, resulting in 34% α-helical structure, 19% β-strands and 47% random coils for the mutant, and in 37% α-helices, 11% β-strands and 52% random coils for the wt. These results are in agreement with the wt crystal structure (PDB ID: 1U2P)^20^. Nonetheless, PtpA D126A had a slightly reduced CD intensity in the 208 nm negative peak compared to the wt, suggesting that there is a small rearrangement in the protein conformation of this mutant. This slight change did not affect the trypsin digestion pattern of PtpA, as the same SDS-PAGE profile was observed after trypsin digestion for 1 to 3 hours (Fig. 1C). In thermal denaturation experiments the wt and mutant PtpA showed a similar profile between 25°C and 40°C, but at higher temperatures a loss of secondary structure was observed for the mutant protein indicating that the Asp 126 molecular contacts contribute to the thermal stability of PtpA wt (Fig. 1D).

The kinetic properties of the PtpA D126A mutant were then examined using *p*NPP, a conventional artificial substrate of phosphatases. An optimal D/A substrate trapping mutant is expected to have a similar *K_m_* but a decreased value of *k_cat_* when compared to the wt enzyme^21^,^22^. In our case this rule was fulfilled as three independent batches of purified PtpA wt and PtpA D126A presented similar values of *K_m_* (7.4 ± 3.1 mM and 5.3 ± 2.3 mM, respectively) while the *k_cat_* for the PtpA D126A (0.04 sec^−1^) was 40-fold lower than that observed for the PtpA wt (1.58 sec^−1^) (Supplementary Fig. 2A). These enzymatic properties reproduced data previously reported for PtpA D126A: a similar *K_m_* value and a marked decrease in *k*_cat _(36-fold)^21^. Furthermore, the obtained K_m_ values of our mutant were comparable to those reported for other PTPs, particularly the PtpA homologue in *Streptomyces coelicolor* A3(2)^28^. In contrast, Bach *et al* found that the D126A mutation caused a total loss of PtpA phosphatase activity, and not a partial loss of function as is expected for this mutant, but still it allowed identifying VPS33B as a putative PtpA substrate^7^. Differences with our results could be due to distinct experimental procedures used to express and/or to purify this mutant. Overall, we obtained a good yield of mycobacterial PtpA D126A mutant that exhibited structural and functional features to act as a useful substrate-trapping mutant for isolating eukaryotic substrates of *Mtb* PtpA.

### Study by SPR of PtpA D126A interactions with macrophage protein components

**Figure 2:**
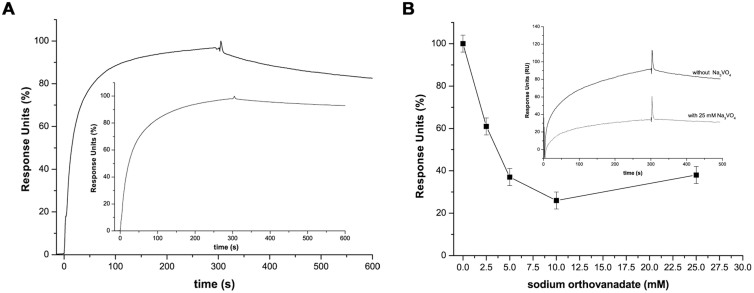
Analysis by SPR of protein-protein interaction. (A) Real time association-dissociation sensorgrams of the macrophage extract with immobilized PtpA D126A. Values are expressed as percentage with 100% corresponding to 90 RU. Inset: association-dissociation sensorgrams corresponding to the interaction of macrophage extract with immobilized PtpA wt. (B) Effect of Na_3_VO_4_ on the association of macrophage components to PtpA D126A. Results are shown as the maximal association response units (RU, expressed as the percentage relative to the response measured without inhibitor) achieved at increasing concentrations of the inhibitor Na_3_VO_4_. Accordingly, a 100% response corresponds to the maximal RU obtained by injection of the macrophage extract without inhibitor. Inset: association-dissociation sensorgrams of macrophage extract mixed with 25 mM sodium Na_3_VO_4_. These sensorgrams are representative of the results obtained in more than three analytical replicates and two biological replicates performed with different batches of purified PtpA and macrophage extracts.

PtpA D126A was covalently immobilized on a CM5 sensor chip to assess by SPR its ability to bind specifically to macrophage components. For that purpose we prepared a macrophage extract using orthovanadate (Na_3_VO_4_) and iodoacetic acid (IAA) to preserve P-Y modifications in proteins. Short-time association-dissociation sensorgrams are shown in Fig. 2A. A significant response was detected during the association phase followed by a slow dissociation, suggesting that the extract contains molecules capable of interacting with PtpA D126A. Since these sensorgrams are comparable to those obtained for PtpA wt (inset Fig. 2A), the PtpA D126A mutant seems to be useful to capture potential PtpA substrates present in this macrophage extract. When two successive short pulses of 1.0 M NaCl were injected, the dissociation rate and signal remained similar to that observed with the running buffer demonstrating that the interaction was resistant to a high ionic strength (Supplementary Fig. 3A). This is in agreement with data showing that several hydrophobic residues in the PtpA active site play a key role in the definition of PtpA substrate specificity^24^. In contrast, pulses of 10 mM glycine pH 2.0 caused a blunt drop of the signal to the baseline, indicating the disruption of putative PtpA D126A complexes at low pH (Supplementary Fig. 3B). The response level was not affected after 4 cycles of regeneration with glycine pH 2.0 (Supplementary Fig. 3C). Furthermore, Na_3_VO_4_, a competitive inhibitor of tyrosine phosphatases, was utilized to analyze whether the active site was involved in the interactions between PtpA D126A and macrophage components. When the inhibitor was mixed with the macrophage extract before injection, a dose dependent decrease in the total response units (RU) was observed (Fig. 2B). This result suggests that the macrophage extract contained components which were capable of binding to PtpA mutant through the active site. Alternatively, PtpA complexes with macrophage components were allowed to form and then pulses of the inhibitor (25 mM) were applied. In this case Na_3_VO_4_ was unable to promote protein components dissociation of PtpA D126A suggesting that the interaction of the PtpA mutant with macrophage components was of higher affinity than with the Na_3_VO_4_. Overall, SPR analysis resulted in a useful tool to define adequate experimental conditions to isolate proteins specifically bound to PtpA D126A during the substrate trapping assay. High salt concentration seems to be a good choice for washing steps to reduce non-specific binding, while low pH would be useful for eluting the proteins bound to PtpA D126A with high affinity.

### Substrate trapping assay

**Figure 3:**
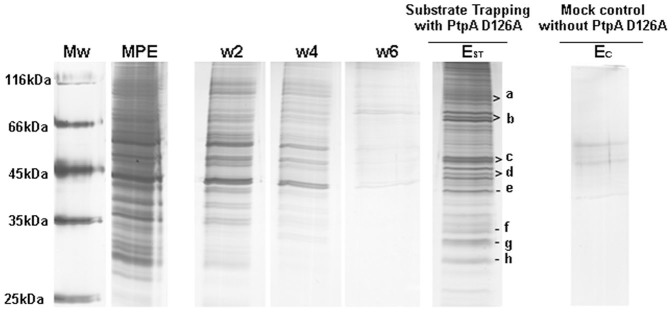
SDS-PAGE analysis of the samples obtained in PtpA D126A substrate trapping assays. Mw, molecular weight marker; MPE, macrophage extract; W2, second wash (with running buffer); W4, fourth wash (with 0.5 M NaCl); W6, sixth wash (with 1.0 M NaCl). E_ST_ represents a pool of the four elutions after the substrate trapping with PtpA D126A, and E_C _a pool of the four elutions after the mock substrate trapping experiment (matrix without immobilized PtpA D126A). These gels are representative of two biological replicates of substrate trapping experiments performed with different batches of purified PtpA and macrophage extracts.

On the basis of SPR studies we designed a substrate trapping assay to pull down novel PtpA substrates from the macrophage extract in which P-Y modifications were preserved. The mutant enzyme was covalently immobilized to an NHS-activated sepharose matrix through the same chemistry used in SPR experiments. As a control we verified that the phosphatase activity was not altered after immobilization (Supplementary Fig. 2C). The binding of PtpA D126A is expected to be uni or bi-punctual since PtpA has only one Lys in addition to the N-terminal group (Supplementary Fig. 2D). The remaining active groups of the matrix were blocked with ethanolamine, an indispensable step to minimize unspecific protein binding. This constitutes an advantage with respect to the His-tag affinity matrix, used in some cases in substrate trapping experiments. The immobilized PtpA D126A was then incubated with the macrophage extract and subsequently washed with high ionic strength solutions (0.5 M and 1 M NaCl) in order to reduce unspecific protein interactions. The putative PtpA D126A partners were firstly recovered by elution with Laemmli sample buffer. Figure 3 shows the SDS-PAGE analysis of the wash and elution samples obtained in PtpA D126A substrate trapping assays. Following six washes of the matrix, almost no protein was detected by silver staining of the gels (lane w_6_). After elution, a band pattern (E_ST_ lane) visibly different from that observed for the input macrophage extract (lane MPE) and washes (w2-w6 lanes), was observed. This suggests that potential partners of PtpA D126A were enriched during the substrate trapping assay. In mock substrate trapping experiments, where the macrophage extracts were loaded on a matrix without immobilized PtpA D126A, only few bands were detected in the elution step (lane Ec). All the E_ST_ and Ec lanes were cut and analyzed by MALDI-TOF MS. Using the PtpA D126A immobilized matrix for substrate trapping, a total of eleven proteins were identified from eight gel pieces of the lane E_ST_ (lettered a-h in Fig. 3), while no proteins were identified in the remaining pieces of the gel. The identified proteins are shown in Table 1. In the mock control experiments (Fig 3, lane Ec) we could not identify any protein by MALDI-TOF MS. However, by using a more sensitive MS approach (nano-LC MS) four of the eleven proteins were identified in the mock control experiments as well (HS90, TBB5, TBA1C, ACTB, Supplementary Table 1). Although we cannot exclude the possibility that some of these four proteins have been specifically enriched during substrate trapping, they were excluded for further analysis as they also bind to the PtpA D126A-free matrix. Moreover, these proteins have been previously found to unspecifically bind to different matrixes^29^,^30^. Taken together these results show that various macrophage components linked to cell energy metabolism were captured using a substrate trapping with PtpA D126A under stringent conditions, including five mitochondrial (ECHA, ATPA, SQRD, MPCP and CY1) and two cytoplasmic proteins (K6PP and HSPB1) (Table 1).

As an alternative approach, proteins associated to PtpA D126A during substrate trapping were eluted by lowering the pH to 2.5. As expected, elution in this condition led to the recovery of minor amounts of proteins than when using Laemmli sample buffer, reason why no bands were detected in gels stained with silver nitrate. Thus, these samples were digested with trypsin and the resulting peptides were identified by nano-LC MS analysis. Due to the increased sensitivity of this approach, the total number of proteins identified was greater but included the proteins identified by MALDI-TOF MS after elution with Laemmli sample buffer (Table 1), as well as other subunits of some of these proteins (ECHB, ATPB, ATPO, ATP5H, Supplementary Table 2).

**Figure 4:**
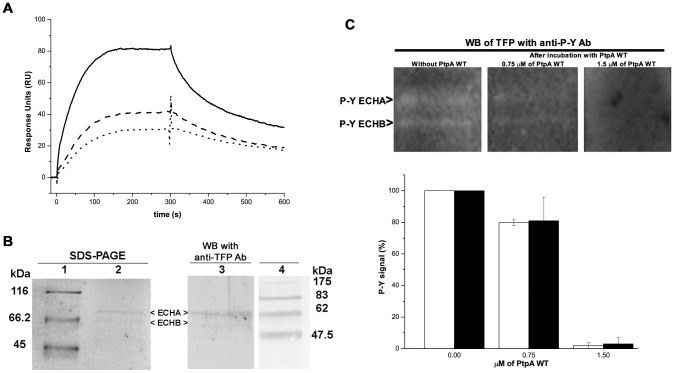
PtpA interacts with and dephosphorylates the TFP (ECHA/ECHB) *in vitro.* (A)Real time association-dissociation sensorgrams of PtpA D126A with immobilized TFP, in absence (continuous line) and in presence of 2.5 mM (dashed line) and 5 mM (dotted line) of Na_3_VO_4_. PtpA D126A was injected at 5 μM, diluted in running buffer at 25°C, during 5 minutes and a flow rate of 30 μl/min. These sensorgrams are representative of the results obtained in two analytical replicates and two biological replicates performed with different batches of TFP and PtpA D126A. (B)Immunodetection of the TFP (ECHA and ECHB subunits). Left, SDS-PAGE stained with Colloidal Coomassie with MW (lane 1) and the TFP enriched fraction (lane 2). Right, Western blot with the anti TFP (ECHA/ECHB) antibody on the same TFP enriched fraction (lane 3), and the MW (lane 4). Immunodetection of P-Y signal in the TFP (ECHA/ECHB) after incubation with 0, 0.75 and 1.5 μM of PtpA wt. The bands were quantified using the GBOX ChemiSystem tool (SynGene), using the raw volume as index of the signal. The P-Y signal obtained after incubation of the TFP with PtpA wt at 0.75 and 1.5 μM was normalized to the signal obtained with PtpA wt at 0 μM (considered as 100%). Error bars represent inter-experimental variability detected in two experiments using two different batches of immunoprecipitated TFP. In the online resource PhosphoSitePlus ECHA is noted as phosphorylated in Y724 and ECHB in Y336, Y342 and Y357^34^.

Independently of the experimental approach used for elution and protein identification, from a total of five biological replicates of substrate trapping (including different batches of purified PtpA D126A and macrophage extracts) only four macrophage proteins were systematically identified and thus considered as PtpA putative substrates: ECHA, SQRD, ATPA and K6PP (Table 2). These proteins were identified with the best scores (from 531 to 2422) and 154 peptide spectrum matches corresponding to 69 different peptide sequences (Supplementary Table 3). On the other hand, these candidates were isolated from macrophage extracts where P-Y modifications were preserved by reversible and irreversible inactivation of PTPs with Na_3_VO_4_ and IAA, respectively. It is known that in the absence of Na_3_VO_4_ the level of P-Y in protein cell extracts is undetectable^31^. However, the use of IAA seems to be of significant help, since we found that the level of P-Y in extracts from Na_3_VO_4_ and IAA treated-macrophages was approximately 29% higher than that detected when the P-Y was preserved only with Na_3_VO_4_ (Supplementay Fig. 4A). Accordingly, when substrate trapping assay was performed with a macrophage extract prepared without IAA, only two of the four candidates were isolated, and identified with a much lower score (ECHA, score 62 and SQRD score 266). This indicates the relevance of the irreversible inhibition of the endogenous PTPs for isolation of P-Y substrates.

As described above PtpA D126A was barely active (see Supplementary Fig. 2), so it is possible that after substrate trapping the isolated proteins were not totally dephosphorylated. In agreement, the analysis by Western blot using an anti P-Y antibody demonstrated the presence of P-Y residues in the proteins recovered by substrate trapping assay (Supplementary Fig. 4B). However, no P-Y containing peptides were detected by MS analysis. A number of factors usually complicate phosphopeptide identification in complex mixtures using nHPLC-MS/MS approach. During MS/MS analysis the release of phosphate group is the main fragmentation at the expense of peptide bond cleavage, resulting in reduced-quality MS/MS spectra and lower-confidence in spectral matching for phosphopeptides^32^,^33^. As shown in Table 3, all the identified PtpA putative substrates contain Tyr residues in the overall sequence and some of them are noted as phosphorylated in the online resource PhosphoSitePlus^34^.

### PtpA interacts with and dephosphorylates the TFP (ECHA/ECHB) *in vitro*

Using immobilized PtpA D126A we isolated the two subunits of the TFP protein (ECHA/ECHB) as putative substrates of PtpA during substrate trapping experiments. To confirm this protein-protein interaction by a contra-experiment, the purified TFP was covalently immobilized on a SPR sensor chip to assess by SPR its ability to bind specifically to PtpA. The association-dissociation sensorgrams shown in Fig. 4A confirmed that PtpA D126A was capable of interacting with TFP and also with PtpA wt (Supplementary Fig. 5). Furthermore, when the competitive inhibitor Na_3_VO_4_ was mixed with the phosphatase before injection, a dose dependent decrease in the total response units was observed (Fig. 4A) suggesting that TFP-PtpA interaction involved the phosphatase active site.

To examine the possibility that phosphorylated TFP (ECHA/ECHAB) acts as a substrate of PtpA, we tested the ability of PtpA wt to dephosphorylate it. Firstly, TFP was purified by immunoprecipitation using a TFP (ECHA/ECHB) antibody (Fig. 4B) and the presence of P-Y was demonstrated by Western blot using an anti-P-Y antibody (Fig. 4C). Equal amounts of the immunoprecipitated TFP were electro-transferred to a membrane and then incubated with recombinant PtpA wt or buffer as a control. The P-Y signal was then evaluated with anti-P-Y antibody. In comparison with controls, a gradual reduction in the phosphorylation levels of both alpha and beta subunit of TFP was observed after incubation with PtpA wt (Fig. 4C). Altogether, these data indicate that, at least *in vitro*, TFP is a bona fide substrate of PtpA.

## Discussion

This work describes an original methodological approach to improve the substrate trapping assay as a tool to capture potential substrates of phosphatases. This approach is mainly based on the analysis by SPR of the interaction between the enzyme and the extract used as a source of potential substrates, which allows selecting stringent conditions for washing and elution steps, thus reducing unspecific protein interactions. In particular, this methodology was applied to the identification of novel substrates of *Mtb* PtpA in human macrophages. For that purpose, we employed the *Mtb* PtpA D126A mutant and extracts of human THP-1 macrophages were P-Y modifications were preserved. The *Mtb* PtpA D126A mutant was expressed, purified and characterized, verifying that it mostly retained the biochemical structural properties of PtpA wt while exhibited enzymatic properties suitable to act as a substrate trapping enzyme. The preservation of P-Y modifications in macrophage proteins was assured by using reversible and irreversible phosphatase inhibitors during macrophage extract preparation. As a result of this improved methodology we identified, as novel potential physiological substrates of *Mtb* PtpA, four macrophage proteins, all linked to cell energy metabolism: TFP (ECHA/ECHB), SQRD, ATPA and K6PP.

VPS33B and the GSK-3, previously identified as substrates of PtpA^7^, were not captured in our substrate trapping assay. This contrasting result should be carefully analyzed because of substantial technical differences between these studies and our work. The VPS33B was isolated by substrate trapping using a PtpA carrying the same D/A mutation but exhibiting no phosphatase activity, a non-desired feature for a D/A substrate trapping mutant. In contrast, in our hands, the purified PtpA D126A showed a similar *K_m_* and a lower (40-fold) k_cat_ than the PtpA wt, which reproduces the functional features reported in a kinetic study of this PtpA mutant^21^. Furthermore, the experimental design used in this work made more rigorous the conditions used during the substrate trapping assay, so that we may have lost components exhibiting lower affinity for PtpA. With respect to GSK-3, it was found to act as a PtpA substrate using a completely different approach where authors performed a Kinome analysis of pre-selected eukaryotic kinases^15^. Thus, the fact that VPS33B and GSK-3 were not captured in our substrate trapping assay is likely a consequence of different experimental approaches and does not invalid the potential of the molecules identified in this work as PtpA substrates. Moreover, one of them, the TFP, was successfully validated by *in vitro* experiments.

TFP, ATPA and SQRD, identified as potential *Mtb* PtpA substrates, are proteins synthesized on cytosolic ribosomes and then translocated to the mitochondria^35^,^36^. Interestingly, specific and marked changes in mitochondrial ultrastructure and function have been recently described in THP-1 macrophages infected with the virulent *Mtb* H37Rv strain^37^. Although the molecular events associated with these alterations have not been elucidated yet, significant changes in the mitochondrial proteome were found. Among others, ECHA, ATPA and SQRD were specifically and strongly modulated (more than 10-fold decrease) by the virulent *Mtb* H37Rv, and, moreover, were no longer detected in the mitochondria^37^. Since PtpA has been localized in the cytosol of *Mtb* infected macrophages^10^, it cannot be ruled out that it may dephosphorylate these proteins altering their activity and/or translocation to the mitochondria. In any case, this hypothesis requires previous validation of ECHA, ATPA and SQRD as PtpA substrates. As a first step in this direction, we demonstrated that TFP (ECHA/ECHB) is a bona fide substrate of PtpA *in vitro* (Fig. 4). TFP plays a key role in β-oxidation of long chain fatty acids, a pathway that provides electrons to the mitochondrial respiratory chain for ATP synthesis^38^. Thus, an eventual TFP mitochondrial deficiency in infected macrophages^37^ suggests that *Mtb* drives macrophage catabolism favoring glycolysis over β-oxidation of long chain fatty acids. This metabolic change may play a role in bacterial survival since the host cellular lipids constitute the primary nutrient source for intracellular *Mtb*^39^. The effect of PtpA on TFP activity and cell distribution needs to be further studied.

The observation that macrophages infected by the virulent *Mtb* H37Rv, but not by the avirulent *Mtb* H37Ra strain, exhibited a pronounced increase in the ATP to ADP ratio^37^, renders the ATPA subunit, a key component of the ATP synthase catalytic core domain F1, an interesting target for further studies^40^,^41^. It is worth to mention that ATP synthase subunits have been reported as targets of several viral proteins acting as pro-viral factors regulating virus replication, transmission and propagation in the host^42^,^43^. The SQRD also represents an interesting candidate as PtpA substrate since this enzyme participates in cell metabolic and microbicidal pathways in cells^44^,^45^,^46^,^47^. This enzyme catalyzes the oxidation of sulfide species (released from the metabolism of sulfur-containing amino acids) to elemental sulfur. The electrons from sulfide oxidation are transferred to the mitochondrial respiratory chain for energy production^45^. Moreover, sulfide oxidation by SQRD contributes to keep sulfide concentration below toxic levels, otherwise sulfide inhibits the cytochrome oxidase (mitochondrial complex IV) interrupting the respiratory chain^46^. On the other hand, in inflammatory macrophages sulfide accumulation likely contributes to down-regulate anti-microbial and pro-apoptotic effects of nitric oxide, as a result of the reaction of sulfide with peroxinitrite^47^. Thus, modulation of SQRD activity and/or cell distribution by *Mtb* may influence both energy metabolism and nitric oxide-dependent microbicidal effects of macrophages.

Finally, since i*n vitro* and *in vivo* studies indicate that *Mtb* infection drives strong metabolic changes in macrophages at the level of glucose consumption^48^ it is worth to study whether PtpA is capable of dephosphorylating the cytoplasmic 6-phosphofructokinase (K6P) which constitutes the most important control step in the mammalian glycolytic pathway^49^,^50^. Indeed, K6P regulates the rate of glycolysis in response to cell energy requirements and is likely linked to the mitochondrial ATP synthesis. Phosphorylation of K6P, which seems to occur mainly in its C-terminal regulatory domain^50^, is involved in the equilibrium between oligomeric forms of the enzyme, in the affinity to substrates and allosteric ligands, in the modulation of the interactions with other proteins, and in its intracellular distribution^50^,^51^,^52^,^53^.

All together, in this work we identified novel components linked to macrophage bioenergetics, particularly TFP, which may be subjected to *Mtb* modulation through PtpA dephosphorylation. Whether *Mtb* PtpA-mediated dephosphorilation of these potential targets affects their activity, cell distribution and/or the metabolic pathways of energy production in macrophages requires further analysis.

## Methods

### Production of recombinant PtpA wt and PtpA D126A and *kinetic characterization*

The coding sequence of PtpA was amplified by PCR from cosmid MTCY427, using appropriate primers with NdeI and HindIII restriction sites. Digested PCR products were ligated with phage T4 DNA ligase (Biolabs) in a pET28 expression vector. The construct expressing the single PtpA mutant D126A was obtained by site-directed mutagenesis using the Quikchange Site-Directed Mutagenesis kit (Stratagene) and the construct verified by DNA sequencing. Transformed *E. coli* BL21 (DE) cells were grown at 15°C in LB medium with 50 μg ml^−1^ kanamycin and protein synthesis induced with 0.5 mM IPTG. The recombinant proteins were purified to homogeneity by metal-affinity (Cu-column) and size exclusion chromatography (SEC). The phosphatase activity was measured using the artificial substrate p-nitrophenyl phosphate (*p*NPP) at 410 nm^9^ and expressed as μM of *p*NP min^−1^ (ε = 7.976 × 10^3^ M^−1^**·**cm^−1^). The kinetic constants K_m_ and V_max_ were calculated using the best fit curve to the Michaelis-Menten equation^54^. For more details see legends of Supplementary Fig 1 and 2.

### Circular Dichroism

Experiments were performed with 25 μM of PtpA D126A or PtpA wt in 20 mM NaH_2_PO_4_ (pH 7.4), 20 mM NaCl. Circular dichroism (CD) spectra were recorded using a JASCO J-715 spectropolarimeter equipped with a thermoelectric sample temperature controller (Peltier system). Data were recorded from 300 to 200 nm at 25°C using a quartz cell with a path length of 10 mm. A total of 5 accumulations were taken for each spectrum with a speed of 50 nm/min, with a resolution of 0.2 nm, responses of 1 s and bandwidth of 1.0 nm. Following background subtraction, all the CD data were converted from CD signal (mdegree) into mean residue ellipticity (deg.cm^2^.dmol^−1^). The secondary structure quantifications were performed with K2d software^27^. Thermal stability of PtpA wt and PtpA D126A was studied by CD spectroscopy. The ellipticity at 222 nm was measured from 25°C to 90°C with an increase step interval of 5°C and a 5 min equilibration at each temperature. Also, renaturation experiments (from 90°C to 25°C) were carried out to ensure reversibility. The change in ellipticity at 222 nm was plotted against the temperature to evaluate thermal stability.

### Preparation of extracts of THP-1 derived macrophages

THP-1 human monocyte-like cells (ATCC TIB-202) were cultured in RPMI 1640 supplemented with 2 mM L-glutamine, 1.5 g/l sodium bicarbonate, 1% sodium pyruvate, 10 mM Hepes pH 7.0, 10% heat-inactivated FCS and maintained at 0.2–1 × 10^6^ cells per ml at 37°C in a humidified 5% CO_2_ atmosphere. When the cultures reached 1 × 10^6^ cells/ml, cells were stimulated and differentiated to macrophages with 50 ng/ml phorbol myristate acetate for 48 hours. To preserve P-Y in proteins, the endogenous PTPs were inactivated treating cells with sodium orthovanadate (Na_3_VO_4_) and iodoacetic acid (IAA)^17^,^55^. Briefly, cells were incubated for 20 min with 100 μM Na_3_VO_4_, washed with PBS and lysed with lysis buffer (25 mM Hepes pH 7.4, 1 mM EDTA, 150 mM NaCl, 1% Triton X-100, 10% glycerol, 1 mM benzamidine, 1 μg/ml trypsin inhibitor, 1 mM PMSF) using a manual homogenizer. Afterwards, a final concentration of 5 mM IAA was added, left on ice for 30 min, and the unreacted IAA inactivated with 10 mM of DTT for 15 min. Lysates were centrifuged at 15,000× *g* for 25 min and cleared extracts filtered (0.22 μm) and stored at −80°C.

### Surface plasmon resonance assays

Experiments were done on a Biacore 3000 GE Healthcare instrument. PtpA D126A was immobilized using standard amine-coupling procedures (Amine Coupling Kit, GE Healthcare) on CM5 sensorchips (GE Healthcare). Ligands immobilization was carried in running buffer 10 mM HEPES pH 7.4, 150 mM NaCl, 3 mM EDTA, 0.005% P20 and 1 mM DTT). After surface activation with 0.05 M NHS (N-hydroxysuccinimide) and 0.2 M EDC (1-ethyl-3-(3-dimethylaminopropyl) carbodiimide) PtpA D126A and PtpA wt were diluted in 10 mM sodium acetate pH 4.5 and injected onto two different sensor chip flow cells at a flow rate of 5 μl/min to reach a final density of 1600 and 400 RU, respectively. Remaining sites were blocked with 1.0 M ethanolamine pH 8.5. Another flow cell was activated and blocked in the same way with no ligand injections and used as reference surface. Sensorchip was washed with 1.0 M NaCl and stabilized with running buffer before sample injections. Injections of macrophage extracts (1.2 μg in protein) were performed in running buffer at 25°C and a flow rate of 10 μl/min unless otherwise stated. Interactions were evaluated by the analysis of the association/dissociation sensorgrams, and their stability was assessed by the injection of two 30 second pulses of 1 M NaCl and by the injection of two 40 second pulses of 10 mM glycine pH 2.0. SPR experiments carried out to study the interaction between TFP (ECHA/ECHB) and the phosphatases (PtpAwt and PtpA D126A) were done in the same way by immobilizing TFP on a CM5 sensorchip to a final density of 360 RU (at flow rate 50 μl/min) and remaining sites blocked using the same procedure. Running buffer used in these experiments was 20 mM Tris-HCl pH 8.0, 50 mM NaCl, 5 mM EDTA, 0.005% P20 and 1 mM DTT and phosphatases injected at flow rate 20 μl/min unless otherwise stated. The effect of incubating with increasing concentrations of Na_3_VO_4_ (2.5–25 mM) was also evaluated. All data processing was carried out using the BIAevaluation 4.1 software (GE Healthcare).

### Substrate trapping using immobilized PtpA D126A

Purified PtpA D126A was covalently coupled to NHS-activated sepharose (GE Healthcare) following instructions provided by the manufacturer. Briefly, the matrix (100 μl) was washed with cold 1 mM HCl, and immediately 300 μg of PtpA D126A diluted in coupling buffer (0.2 M NaHCO_3_, 0.5 M NaCl pH 8.3) were added and incubated 16 hs at 4°C. Unreacted groups of the matrix were blocked overnight at 4°C with 0.5 M ethanolamine pH 8.3, 0.5 M NaCl. Then, the matrix was washed with 0.1 M Tris-HCl pH 8.0 and 0.1 M acetate buffer pH 4.5, 0.5 M NaCl. In parallel, as a control for unspecific binding in pull-down assays the same amount of matrix was incubated in coupling buffer without PtpA D126A, and then blocked with ethanolamine following the same protocol, and this control was defined as the mock substrate trapping (see Supplementary Fig. 2). For each substrate trapping assay, 100 μl of the matrix with or without immobilized PtpA D126A was incubated with 5 mg of macrophage extract diluted to 0.17 mg/ml in SPR running buffer containing 1 mM benzamidine, 1 mM PMSF, 1 μg/ml SBTI during 1 h at 4°C with gentle end-over-end agitation. The matrix was collected by low-speed centrifugation (1,000× *g*) and then washed twice and sequentially with 100 μl of running buffer and running buffer containing 0.5 M and 1.0 M NaCl at 4°C. The retained proteins were eluted using one of the two approaches described below. In the first case, 50 μl of Laemmli sample buffer were added to the matrix and then boiled for 5 min. All washes and elutions were analyzed by SDS-PAGE followed by silver staining. This approach was performed on two biological replicates. The lanes corresponding to the eluted proteins were cut in 2 mm bands and each gel piece was analyzed by MALDI-TOF MS. In the second approach, to reproduce the elution conditions of the SPR assays, proteins were eluted with successive additions of 50 μl of 10 mM glycine pH 2.5 followed by an immediate neutralization with 2 μl of 1 M Tris-HCl pH 7.5, and stored at −20°C until analysis by Nano-LC-MS/MS. This approach was performed on three biological replicates. Each biological replicate was performed using a different batch of the macrophage extract and purified PtpA D126A.

### Mass spectrometry

For MALDI-TOF MS analysis proteins were in-gel digested with trypsin (sequence grade, Promega) as previously described^56^. Peptides were extracted from gels using aqueous 60% acetonitrile (ACN) containing 0.1% TFA and concentrated by vacuum drying. Prior to MS analysis, samples were desalted using C18 reverse phase micro-columns (Omix®Tips, Varian) and eluted directly onto the MALDI sample plate with matrix solution α-cyano-4-hydroxycinnamic acid in 60% ACN containing 0.1% TFA. Mass spectra of peptides mixtures were acquired in a 4800 MALDI TOF/TOF instrument (ABi Sciex) in positive reflector mode and were externally calibrated using a mixture of peptide standards (Applied Biosystems). Collision-induced dissociation MS/MS spectra of selected peptides ions were acquired. Proteins were identified with measured *m/z* values in MS and MS/MS acquisition modes and using the MASCOT search engine (Matrix Science, http://www.matrixscience.com) in the Sequence Query search mode. The following search parameters were used for searching the NCBInr database (NCBInr 20130721): taxonomy *Homo sapiens*; protein mass was unrestricted; monoisotopic mass tolerance, 0.05 Da; fragment mass tolerance, 0.3 Da; partial methionine oxidation, cysteine carbamidomethylation and tyrosine phosphorylation as variable modifications; and one missed tryptic cleavage allowed. Significant protein scores (p < 0.05) and at least one peptide ion significant score (p < 0.05) per protein were used as criteria for positive identification.

For nano-LC-MS-analysis, proteins in bands were in-gel digested as described above. In the case of liquid samples (50 μl), proteins were digested with sequencing-grade trypsin (0.25 μg, 12 h at 37°C), desalted, dried by vacuum and resuspended in 20 μl of 0.1% formic acid (v/v) in water. Samples were injected into a nano-HPLC system (Proxeon easynLC, Thermo Scientific) fitted with a reverse-phase column (easy C18 column, 3 μm; 75 μm ID × 10 cm; Proxeon, Thermo Scientific) and peptides were separated with a linear gradient of acetonitrile 0.1% formic acid (0–60% in 60 min) at a flow rate of 400 nL/min. Online MS detection/analysis was carried out in the LTQ Velos nano-ESI-linear ion trap instrument (Thermo Scientific) in a data-dependent mode (full scan followed by MS/MS of the top 5 peaks in each segment, using an exclusion dynamic list). Proteins were identified by searching the SwissProt database (November 2012), taxonomy *Homo sapiens*, using the following parameters in the MS/MS ion search mode of Mascot search engine: peptide tolerance 1.5 Da, MS/MS tolerance 0.8 Da, and cysteine carbamidomethylation, methionine oxidation and tyrosine phosphorylation as the allowed variable modifications. The significance limit for protein identification was set at p < 0.01 and an ion cut off > 40. Only proteins identified with two or more peptides were considered positively identified. The list of potential PtpA partners was elaborated by manually removal of all the proteins identified in mock control experiments. Only proteins identified in all substrate trapping (five biological replicates), independently of the approach of protein elution and MS analysis, were considered as potential substrate.

### *In vitro* dephosphorylation of ECHA with purified PtpA wt

The TFP (ECHA/ECHB) was obtained from macrophage extracts by immunoprecipitation with anti-TFP MAb. Briefly, the anti-TFP MAb (8 μg, ab110302, MitoSciences) was firstly covalently cross-linked to anti-mouse IgG Ab on the beads (100 μl, 11201D, Life technologies) using BS3 (Sigma). Then, beads were washed and incubated with 500 μg of macrophage extract, washed and bound proteins eluted with citrate pH 2.6 (2 × 100 μl) and neutralized immediately. Samples were resolved by SDS-PAGE and either stained with colloidal Coomassie or transferred to nitrocellulose during 1 h at 100 V. Nitrocellulose was blocked for 16 hs at 4°C, washed twice with TBS-T and subsequently incubated with anti-TFP MAb at 2 μg/ml at RT. After washing, membranes were incubated (1 h at RT) with anti-mouse Ab conjugated with alkaline phosphatase (1/30000, Sigma-Aldrich A3562). The reaction was developed with a NBT/BCIP solution (Sigma). The presence of the TFP was confirmed by mass spectrometry. To examine if phosphorylated TFP is a suitable substrate for PtpA, we used the protocol described by Najarro et al^57^. Equal amounts of the purified TFP were resolved by SDS-PAGE, transferred to nitrocellulose, blocked and incubated (30°C for 1 h) with purified PtpA wt (at final concentrations of 0.75 and 1.5 μM) or the phosphate buffer as a control. Afterward, membranes were washed in TBS-T and probed for P-Y levels with anti-P Tyr Ab (Invitrogen #136600) at 0.6 μg/ml. Blots were then incubated with horseradish peroxidase (HRP)-linked anti-mouse (Sigma-Aldrich A4416, 1:10000) secondary antibody for 1 h at RT. After four washes with TBS-T, one wash with TBS, the reaction was developed with Super Signal West Pico Chemiluminiscent Substrate (Thermo Scientific). Immunoreactive bands were visualized using the GBOX ChemiSystem tool (SynGene).

## Supplementary Material

Supplementary Information Supplementary information

## Figures and Tables

**Table 1:** Proteins identified by MALDI-TOF-MS in gel bands of PtpA D126A substrate trapping assays

Band Name^a^	Protein name	Protein accession	Mass (Da)	Mascot score^b^	No. of matched sequences^b^	No. of peptide sequences^b^	Sequence coverage^b^ (%)	Peptide sequences confirmed by MS/MS
**a**	Heat shock protein HSP-90 beta	HS90_HUMAN^f^	83242	134	8	7	13	**IDIIPNPQERGVVDSEDLPLNISR**
**b**	Trifunctional enzyme subunit alpha, mitochondrial	ECHA_HUMAN	82947	127	21	16	28	**TLQEVTQLSQEAQRTVLGTPEVLLGALPGAGGTQR**
	6-phosphofructokinase, platelet type	K6PP_HUMAN	85542	118	24	19	31	**YLEEIATQMRAIGVLTSGGDAQGMNAAVR**
**c**	ATP synthase subunit alpha, mitochondrial	ATPA_HUMAN	59714	128	10	9	26	**EAYPGDVFYLHSRTGAIVDVPVGEELLGREVAAFAQFGSDLDAATQQLLSR**
	Tubulin beta-5 chain^c^	TBB5_HUMAN^f^	49640	261	26	21	61	**FPGQLNADLRAILVDLEPGTMDSVRISEQFTAMFR**
	Tubulin alpha-1C chain^d^	TBA1C_HUMAN^f^	49863	163	18	16	47	**AVFVDLEPTVIDEVR**
**d**	Sulfide quinone oxidoreductase, mitochondrial	SQRD_HUMAN	49929	188	22	19	49	**IMYLSEAYFRIMYLSEAYFR+Oxid(M)YADALQEIIQER GYWGGPAFLR**
**e**	Actin, cytoplasmic^e^	ACTB_HUMAN^f^	41710	82	4	4	15	**SYELPDGQVITIGNER**
**f**	Phosphate carrier protein, mitochondrial	MPCP_HUMAN	39933	76	7	6	18	**IQTQPGYANTLR**
**g**	Cytochome C1, mitochondrial	CY1_HUMAN	35399	77	3	2	9	**AANNGALPPDLSYIVR+Deamidated NQ**
**h**	Heat Shock protein beta 1	HSPB1_HUMAN	22768	167	5	5	29	**LFDQAFGLPRLATQSNEITIPVTFESR**

^a^Band name corresponds to the gel pieces indicated in the SDS-PAGE shown in Fig. 3.

^b^For each protein the value of score, number of matched and peptide sequences indicated is the best value obtained from two biological replicates.

^c^TBB3_HUMAN, TBB2A_HUMAN and TBB4B_HUMAN were also identified with the same set of peptides.

^d^TBA1A_HUMAN, TBA1B_HUMAN were also identified with the same set of peptides.

^e^ACTG_HUMAN was also identified with the same set of peptides.

^f^Proteins identified in mock substrate trapping using the more sensitive approach of Nano LC-MS.

**Table 2:** Proteins indentified as putative PtpA partners

Biological process^a^	Protein accession	Protein name	Mascot score^b^	Mass (Da)	No. of matched ions^b^	No. of peptide sequences^b^
*Lipid metabolism, fatty acid beta-oxidation*	ECHA_HUMAN	Trifunctional enzyme subunit alpha, mitochondrial	2422	82947	63	23
*Sulfide oxidation, using sulfide:quinine oxidoreductase*	SQRD_HUMAN	Sulfide:quinone oxidoreductase, mitochondrial	705	49929	28	10
*Respiratory electron transport chain*	ATPA_HUMAN	ATP synthase subunit α, mitochondrial	531	59714	16	7
*Glycolysis*	K6PP_HUMAN	6-phosphofructokinase, platelet type	922	85542	47	15

^a^ Only the main biological processes with traceable author statement are shown, form UniProt (http://www.uniprot.org) database (released on January 2014)^58^.

^b^ The value of score, number of matched ions and peptide sequences is the best value obtained.

**Table 3:** Residues of Tyr and P-Y in the potential PtpA substrates

Protein accession	Protein name	Targeting signal^a^	Tyr in the targeting signal	Tyr in the human overall sequence
ECHA_HUMAN	Trifunctional enzyme subunit alfa	>sp|P40939|1–35 MVACRAI-GILSRFSAFRILRS-RGYICRNFTGSSALL	Y24	Y24, Y43, Y158, Y239, Y271, Y283, Y298, Y320, Y343, Y435, Y499, Y546, Y637, Y639, **Y724**, Y736, Y740, Y762
SQRD_HUMAN	Sulfide quinone oxidoreductase	>sp| Q9Y6N5 1–66 MVPLVAVV-SGPRAQLFAC-LLRLGTQQVG-PLQLHTGASH-AARNHYEVLV-LGGGSGGITM-AARMKRK	Y44	Y44, Y82, Y138, Y140, **Y151**, Y170, Y210, Y215, Y242, **Y259**, Y289, Y228, Y373, Y376, Y385, Y395, **Y415**, Y426, Y434
ATPA_HUMAN	ATP synthase subunit α	>sp|P25705|1–43 MLSVRVAAA-VVRALPRR-AGLVSRNAL-GSSFIAARNF-HASNTHL	N	**Y243**, **Y246**, Y271, Y287, Y291, **Y299**, Y311, Y321, **Y337**, **Y343**, Y380, Y401, **Y440**, Y476, Y489, Y495
K6PP_HUMAN	6-phosphofructokinase platelet type	NA	NA	Y52, Y58, Y61, Y56, Y162, Y164, Y223, Y298, Y394, **Y447**, **Y487**, Y512, **Y586**, Y589, Y604, **Y645**, **Y651**, **Y654**, Y764, Y768

^a^Extracted from the online resource UniProt^58^ or predicted with MitoProt II^59^. In bold, tyrosine residues noted up to now as phosphorylated in the online resource PhosohoSitePlus (http://www.phosphosite.org)^34^. N: None tyrosine in the mitochondrial targeting signal. NA: Not Apply.
