# Supplementary material for: New potential eukaryotic substrates of the mycobacterial protein tyrosine phosphatase PtpA: hints of a bacterial modulation of macrophage bioenergetics state
Source: Sci Rep. 2015 Mar 6;5:8819. doi: 10.1038/srep08819 (PMC5390082; doi:10.1038/srep08819)
Supplement: Supplementary Information [file srep08819-s1.pdf]

SUPPLEMENTARY FIGURES AND LEGENDS

New potential eukaryotic substrates of the mycobacterial protein tyrosine phosphatase PtpA: hints of a bacterial modulation of macrophage bioenergetics state.

Mariana Margenat, Anne-Marie Labandera, Magdalena Gil, Federico Carrion, Marcela Purificação, Guilherme Razzera, María Magdalena Portela, Gonzalo Obal, Hernán Terenzi, Otto Pritsch, Rosario Durán, Ana María Ferreira, Andrea Villarino.

Supplementary Figure 1. Expression and purification of PtpA D126A.

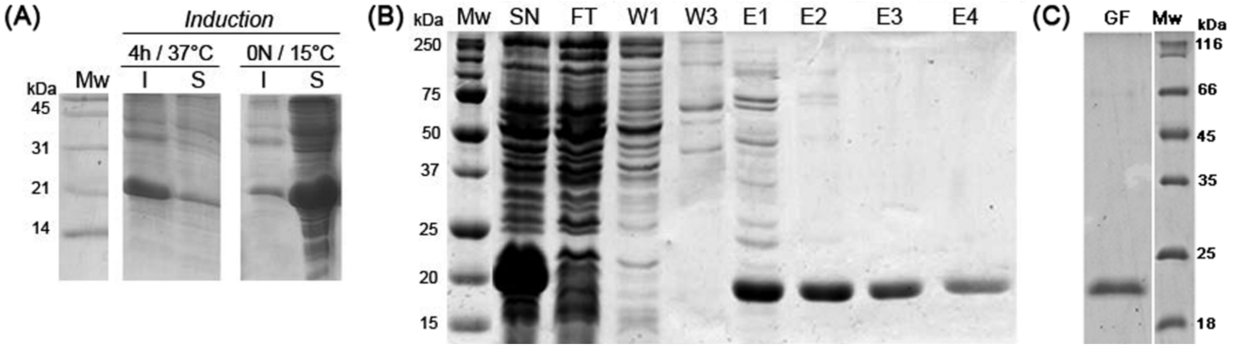

(A) SDS-PAGE (15% gel) analysis of PtpA expression in *E. coli* cells: insoluble fraction (I) and soluble fraction (S) after different conditions of induction.

(B) SDS-PAGE (15% gel) analysis showing the steps of PtpA D126A purification by affinity chromatography: supernatant applied to the Chelating Sepharose Fast Flow matrix (SN); flow through (FT); washes (W1-W3); four successive eluates with 300 mM imidazole (E1-E4).

(C) SDS-PAGE (15% gel) analysis showing PtpA D126A after SEC onto a Superdex 200 16/60 preparative grade column (GE Healthcare). Mw, molecular weight marker. All gels were stained with Coomassie Brilliant Blue R-250. Results are representative of three biological replicates performed with different batches of purified protein.

Details of the methodology: the coding sequence of PtpA from *Mtb* was amplified by PCR from the cosmid MTCY427, using a forward primer including NdeI restriction site (5'-GGGAGGCGCCATATGTCTGATCC-3') and a reverse primer containing a HindIII restriction site (5'-CCTGGCTCAATTCGAAGGCGGATC-3'). The oligonucleotide used to mutate aspartate 126 to alanine in PtpA was 5'-GCTCGATGTCGAGGCTCCCTACTATGGCG-3' (the underlined bases indicate the alteration to encode alanine rather than aspartate). *Escherichia coli* strain BL21(DE3) was separately transformed with pET28a-PtpA WT or pET28a-PtpA D126A plasmid to produce recombinant His-tagged proteins. Transformants were grown overnight in 10 ml of Lysogeny broth (LB) containing 50 μg ml^{-1} kanamycin at 37 °C. For protein expression, 0.5 ml of overnight culture were transferred into 500 ml of LB and grown at 37°C with agitation (140 rpm) up to the end of exponential phase of growth, corresponding to an optical density of 0.6 at 600 nm. Then, for induction of protein synthesis a final concentration of 0.5 mM isopropyl-β-D-thiogalactopyranoside (IPTG) was added. To optimize the production of soluble recombinant proteins cultures were incubated overnight at 15 °C or 4 h at 37 °C. Cells were then harvested by centrifugation at 5,000 x*g* for 30 min, and the pellet was resuspended in cold lysis buffer (20 mM Tris-HCl pH 8.0, 0.5 M

NaCl, 10 mM imidazole, 10% glycerol) containing EDTA-free protease inhibitor cocktail (Amersham Biosciences). Cells were then lysed by sonication on ice (Fisher Scientific Model 60 Sonic Dismembrator) and subsequently centrifuged at 12,000 x*g* for 1 h at 4 °C. His-tagged recombinant proteins were purified from the supernatant by immobilized Cu-affinity chromatography. Briefly, the supernatant was incubated for 40 min at room temperature under agitation with 3 ml of Chelating Sepharose Fast Flow (GE Healthcare, Uppsala, Sweden) previously activated with 0.1 M $CuSO_4$ and equilibrated in lysis buffer, following the manufacturer's instructions. The unbound protein was removed with three washes (W1, W2, W3) of five bed volumes of lysis buffer containing 10 mM, 20 mM, and 30 mM imidazole, respectively. Then, the recombinant protein bound to the matrix was eluted in five steps with two bed volumes of lysis buffer containing 300 mM imidazole. Fractions were analysed by SDS-PAGE and those containing purified recombinant protein were pooled and dialyzed at 4 °C in four steps for 2 h against the elution buffer with decreasing concentrations of imidazole (150 mM, 75 mM, 35 mM and without imidazole). The last dialysis buffer contained 20 mM Tris-HCl pH 8.0, 50 mM NaCl, 5 mM EDTA, 10% glycerol, 5 mM DTT. Then, proteins were concentrated by ultrafiltration with a 10 kDa pore membrane (Amicon Ultra-15 Millipore) and stored at -20 °C. Protein concentration was determined by Bradford assay using bovine serum albumin as standard. To further purify PtpA WT and PtpA D126A and separate aggregate species from monomeric PtpA, a further step of purification by size exclusion chromatography (SEC) was performed in an AKTA Basic system (GE Healthcare). For preparative SEC, samples of 5 ml of dialyzed protein (5 mg) were injected in a Superdex 200 16/60 preparative grade column (GE Healthcare). Elution was carried out with two bed column volumes of the equilibration buffer (last dialysis buffer) and fractions containing the recombinant protein PtpA were pooled, concentrated by centrifugation, supplemented with 20 mM DTT final concentration and the aliquots stored at -20ºC. The amount of pure PtpA, as mg per liter of culture was expressed as mean ± standard deviation of three independent experiments of protein purification.

Supplementary Figure 2.

(A)

	V_{max} (µmol *p*NP min^{-1} mg^{-1})	k_{cat} (sec^{-1})	K_m (mM)
PtpA WT	4.75 ± 0.71	1.58 ± 0.24	7.4 ± 3.1
PtpA D126A	0.12 ± 0.08	0.04 ± 0.02	5.3 ± 2.3

(B)

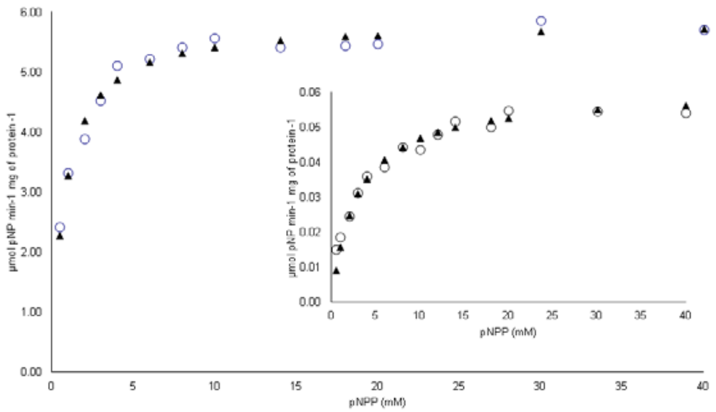

(C)

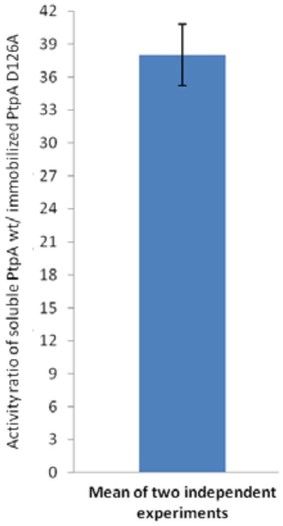

(D)

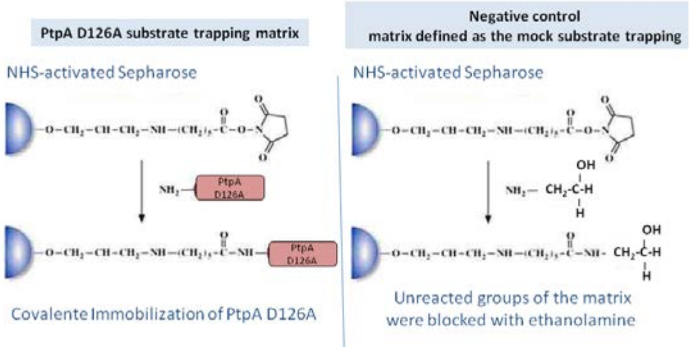

His-tag-PtpA D126A

MGSSHHHHHHSSGLVPRGSVSDPLHVTFVCTGNICRSPMAEKMFAQQLRHRG
LGDAVRVTSAGTGNWHVGSCADERAAGVLRAHGYPTDHRAAQVGTEHLAAD
LLVALDRNHARLLRQLGVEAARVRMLRSFDPRSGTHALDVEAPYYGDHSDFEEV
FAVIESALPGLHDWVVDERLARNGPS

(A) Kinetic parameters values of *M. tuberculosis* PtpA wt and PtpA D126A expressed as mean ± standard deviation of three independent experiments realized with different batches of purified protein.

(B) One example of the Michaelis-Menten representation of one batch of purified PtpA wt and PtpA D126A. White circles represent the experimental data and black circles represent the best fit curve to the Michaelis-Menten equation used to determine the K_m and V_{max} values**.** Reaction rates of PtpA wt and PtpA D126A (inset) are expressed as specific activity (µmoles *p*NP min^{-1} mg^{-1} of protein). The k_{cat} was calculated with the equation $k_{cat} = V_{max}/E_o$, with V_{max} values as µM *p*NP sec^{-1} and E_o as µM of PtpA wt or PtpA D126A.

(C) Activity ratio of the soluble PtpA wt respect to the immobilized PtpA D126A. The activity of equal amount of soluble PtpA wt and immobilized PtpA D126A was evaluated by an endpoint assay in conditions of V_{max} (using 30 mM of *p*NPP). The whole amount of protein offered to the matrix was immobilized. In these conditions, if all PtpA D126A activity is retained after immobilization, the activity ratio of soluble PtpA wt/ immobilized PtpA D126A is expected to be 40. The obtained result confirmed that the immobilized PtpA D126A retained its activity. The results represent the mean ratio from two independent experiments of immobilization.

(D) Diagram showing the strategy used for covalent immobilization of PtpA D126A and the matrix used as a negative control. We also showed the sequence of PtpA D126A indicating in red the only lysine (K) residue, involved in the unipuntual immobilization of the phosphatase to the NHS activated sepharose.

Supplementary Figure 3. Real time association-dissociation sensograms obtained by SPR of macrophage protein extract.

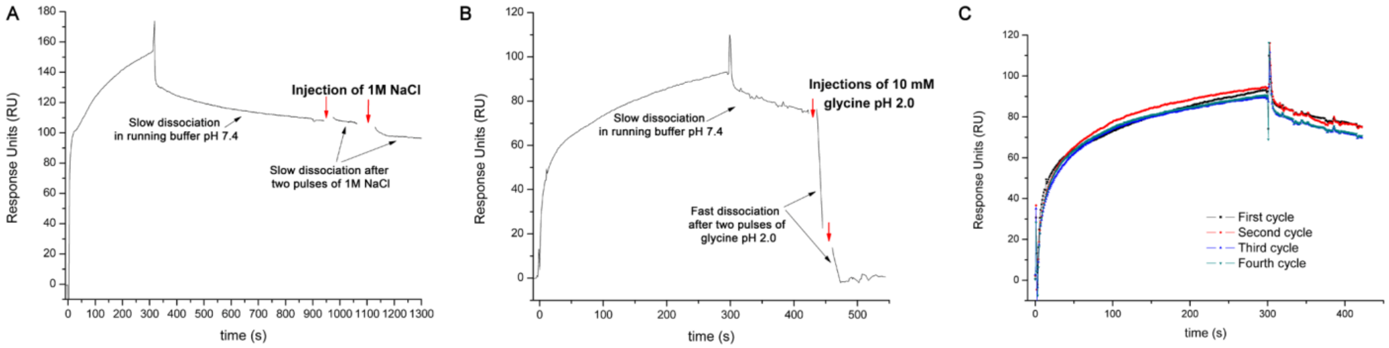

(A) Effect in the dissociation rate of two 30 second pulses of 1.0 M NaCl.

(B) Effect in the dissociation rate after two 40 second pulses of 10 mM glycine pH 2.0.

(C) Association-dissociation curves after four cycles of injection of macrophage protein extract followed by 10 mM glycine pH 2.0.

Supplementary Figure 4. Immunodetection of tyrosine phosphorylated proteins.

(A)

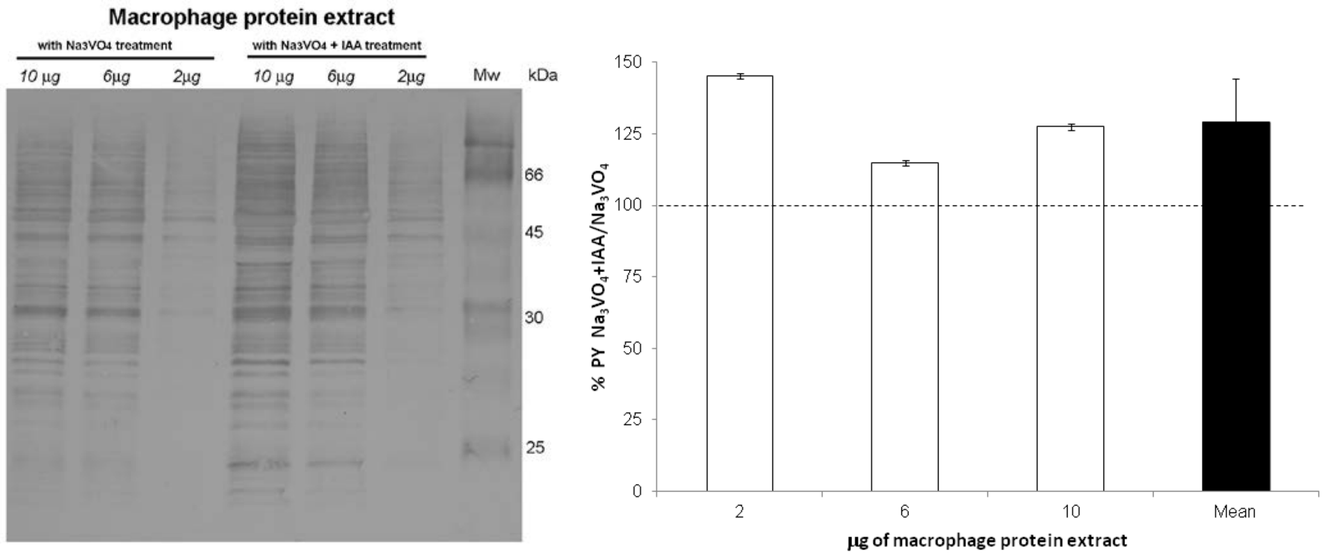

(B)

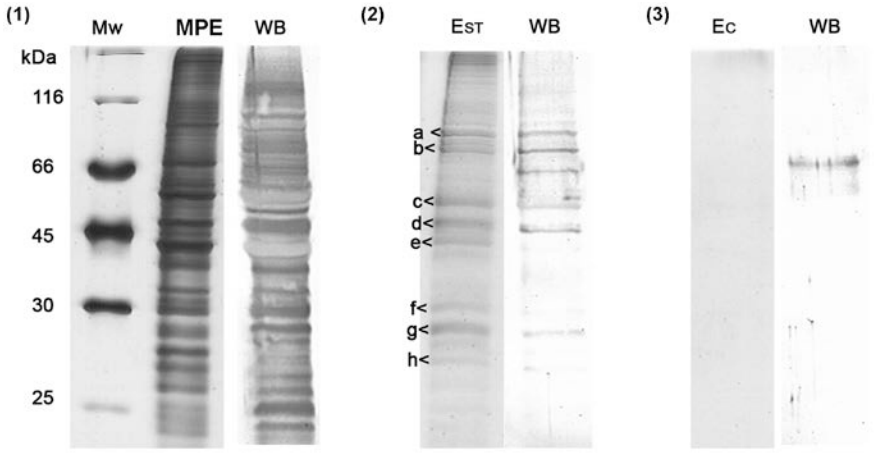

(A) Immunodetection of tyrosine phosphorylated proteins in P-Y enriched protein extracts. Protein concentration was determined by Bradford assay. Samples containing 2, 6 and 10 µg of the macrophage protein extract treated with Na_3VO_4 or with Na_3VO_4 + IAA were separated by SDS-PAGE and transferred to nitrocellulose membranes during 1 h at 100V. The membrane was blocked with Membrane Blocking Solution (Invitrogen) 16 hs at 4 °C and then washed twice with Tris buffered saline pH 7.4, 0.1% tween 20 (TBS-T) followed by incubation with PY Plus Mouse Anti-P Tyr Ab (Invitrogen #136600) at 0.6 µg/mL in TBS-T for 1 hour at RT with gentle agitation. After three washes with TBS-T the membrane was incubated for 1 h at RT with anti-mouse antibody conjugated with alkaline phosphatase (Calbiochem) diluted 1/400 in TBS-T. After washes with TBS-T the reaction was developed with a NBT/BCIP solution (Sigma). The graph shows the P-Y levels (%) of the macrophage protein extract treated with Na_3VO_4 + IAA respect to the extract treated with Na_3VO_4, quantified with the GBOX ChemiSystem tool (SynGene) using the raw volume as index of the signal. The analysis shows that the treatment with Na_3VO_4 + IAA determined an increase of 29% in the level of P-Y signal.

(B) SDS-PAGE and immunodetection of P-Y proteins:

(1) macrophage protein extract (MPE) and the respective Western blot (WB);

(2) fraction eluted in the substrate trapping with PtpA D126A (E_{ST}) and the respective WB. Letters a to h indicate bands which were cut and proteins identified by MALDI-TOF MS (Table 1);

(3) fraction eluted in the mock substrate trapping using the matrix without immobilized PtpA D126A (E_C) and the respective WB. The PY signal observed in the WB does not correspond to a visible band in the silver stained gel.

Supplementary Figure 5. Analysis by SPR of the interaction between PtpA wt with TFP.

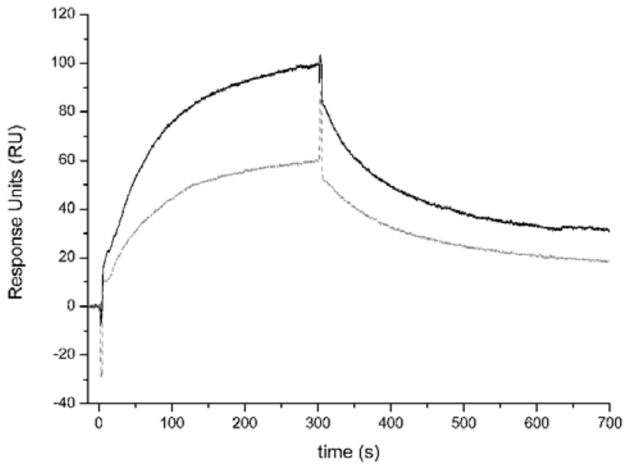

Real time association-dissociation sensograms of PtpA wt with immobilized TFP. PtpA wt injections of 20 µg (black line) and 10 µg (gray line) diluted in running buffer at 25°C and a flow rate of 40 µl/min.

Supplementary Table 1. Proteins identified by Nano-LC MS in mock control experiments

Protein accession	Protein name
K2C1_HUMAN	Keratin, type II cytoskeletal 1
K1C9_HUMAN	Keratin, type I cytoskeletal 9
K1C10_HUMAN	Keratin, type I cytoskeletal 10
K22E_HUMAN	Keratin, type II cytoskeletal 2 epidermal
K2C6B_HUMAN	Keratin, type II cytoskeletal 6B
K1C14_HUMAN	Keratin, type I cytoskeletal 14
K1C16_HUMAN	Keratin, type I cytoskeletal 16
K2C5_HUMAN	Keratin, type II cytoskeletal 5
TBA1A_HUMAN	Tubulin alpha-1A chain
TBA1B_HUMAN	Tubulin alpha-1B chain
TBA1C_HUMAN	Tubulin alpha-1C chain
TBB4A_HUMAN	Tubulin beta-4A chain
TBB4B_HUMAN	Tubulin beta-4B chain
TBB5_HUMAN	Tubulin beta chain
TBB3_HUMAN	Tubulin beta-3 chain
ACTA_HUMAN	Actin, aortic smooth muscle
ACTB_HUMAN	Actin, cytoplasmic 1
ACTC_HUMAN	Actin, alpha cardiac muscle 1
ACTG_HUMAN	Actin, cytoplasmic 2
ACTH_HUMAN	Actin, gamma-enteric smooth muscle
ACTS_HUMAN	Actin, alpha skeletal muscle
HSP7C_HUMAN	Heat shock cognate 71 kDa protein
HS90_HUMAN[f]	Heat shock protein HSP-90 beta
VDAC1_HUMAN	Voltage-dependent anion-selective channel protein 1
RS3_HUMAN	40S ribosomal protein S3
POTEF_HUMAN	POTE ankyrin domain family member F
POTEE_HUMAN	POTE ankyrin domain family member
TRY1/TRY2/TRY3/TRY6_HUMAN	Trypsin-1/ Trypsin-2/ Trypsin-3/ Trypsin-6
CDK4_HUMAN	Cyclin-dependent kinase 4
MESP2_HUMAN	Mesoderm posterior protein 2
EPCAM_HUMAN	Epithelial cell adhesion molecule
RECK_HUMAN	Reversion-inducing cysteine-rich protein with Kazal motifs

Supplementary Table 2. Proteins identified by Nano-LC-MS in the substrate trapping experiments.

Biological process[a]	Protein accession	Protein name	Mascot score[b]	Mass (Da)	No. of matched ions[b]	No. of peptide sequences[b]
Proteins identified in three biological replicates of substrate trapping						
Lipid metabolism, fatty acid beta-oxidation	ECHA_HUMAN	Trifunctional enzyme subunit alpha, mitochondrial	2422	82947	63	23
Sulfide oxidation	SQRD_HUMAN	Sulfide:quinone oxidoreductase, mitochondrial	705	49929	28	10
Respiratory electron transport chain	ATPA_HUMAN	ATP synthase subunit α, mitochondrial	531	59714	16	7
Glycolysis	K6PP_HUMAN	6-phosphofructokinase, platelet type	922	85542	47	15
	K6PL_HUMAN	6-phosphofructokinase, liver type	567	84964	20	9
Proteins identified in two biological replicates of substrate trapping						
Lipid metabolism	GLCM_HUMAN	Glucosylceramidase	806	59678	19	12
	ECHB_HUMAN	Trifunctional enzyme subunit beta, mitochondrial	211	51262	5	4
Signal transduction	CAP1_HUMAN	Adenylyl cyclase-associated protein	485	51869	12	6
	ZYX_HUMAN	Zyxin	232	61238	4	3
Respiratory electron transport chain	CY1_HUMAN	Cytochrome c1, heme protein, mitochondrial	333	35399	11	4
	ATPB_HUMAN	ATP synthase subunit β, mitochondrial	274	56525	8	4
	ATPO_HUMAN	ATP synthase subunit O , mitochondrial	251	23263	6	4
	ATP5H_HUMAN	ATP synthase subunit d, mitochondrial	124	18480	3	3
Protein folding, transport and/or synthesis	GRP78_HUMAN	78 kDa glucose-regulated protein or Heat shock 70 kDa protein	929	72288	21	13
	GRP75_HUMAN	75 kDa glucose-regulated protein or Heat shock 70 kDa protein 9	199	73635	4	3
	CH60_HUMAN	60 kDa heat shock protein, mitochondrial	458	61016	8	7
	HSPB1_HUMAN	Small heat shock protein Hsp25/27	210	22768	4	3
	COPE_HUMAN	Coatomer subunit epsilon	131	34460	3	2
	EF1A1_HUMAN[c]	Elongation factor 1-alpha 1	258	50109	9	5
Nucleic acid binding	SSBP_HUMAN	Single-stranded DNA-binding protein, mitochondrial	312	17249	6	3

[a] Only the main biological processes of each protein with traceable author statement are shown, form UniProt (http://www.uniprot.org) database (released on January 2014) [60].
[b] The value of score, number of matched ions and peptide sequences indicated is the best value obtained from three biological replicates
[c] EF1A3_HUMAN was also identified with the same set of peptides
The MPCP_HUMAN (identified by MALDI-TOF-MS in gel bands) was excluded from the table as it was identified by Nano-LC-MS only with one peptide (in two experiments).

Supplementary Table 3. Nano-LC-MS/MS and Mascot search results for putative PtpA substrates.

ECHA_HUMAN Trifunctional enzyme subunit alpha, mitochondrial

Score	Mass	Matched ions	Peptide sequences	Dupes	m/z obs	M exp	M calc	Delta	Miss	Pept score	Rank	U	Peptide
2426	82947	63	23										
				1	418.47	834.92	833.44	1.48	0	44	1	U	R.GQQQVFK.G
					435.15	868.29	867.44	0.84	0	43	1	U	K.HVAEDLGK.V
				1	470.62	939.23	939.47	-0.24	0	51	1	U	K.FGELVMTK.E + Oxidation (M)
				1	521.38	1040.75	1039.53	1.22	0	60	1	U	R.FVDLYGAQK.I
				3	559.74	1117.46	1117.60	-0.13	0	79	1	U	K.DTSASAVAVGLK.Q
				2	602.23	1202.45	1202.60	-0.15	0	65	1	U	K.GFYIYQEGVK.R
				1	660.15	1318.28	1317.72	0.56	0	75	1	U	K.MQLLEIITTEK.T
				1	668.06	1334.11	1333.72	0.40	0	81	1	U	K.MQLLEIITTEK.T + Oxidation (M)
				1	696.66	1391.30	1391.69	-0.40	0	73	1	U	K.VIGMHYFSPVDK.M
				1	705.52	1409.03	1407.69	1.34	0	72	1	U	K.VIGMHYFSPVDK.M + Oxidation (M)
				1	761.52	1521.03	1520.74	0.29	0	106	1	U	K.DLNSDMDSILASLK.L
					781.64	1561.27	1560.78	0.49	0	98	1	U	K.MVGVPAALDMMLTGR.S
				1	789.42	1576.83	1576.78	0.05	0	67	1	U	K.MVGVPAALDMMLTGR.S + Oxidation (M)
				1	789.58	1577.14	1576.78	0.37	0	90	1	U	K.MVGVPAALDMMLTGR.S + Oxidation (M)
					789.62	1577.23	1576.79	0.44	0	117	1	U	R.FGGGNPELLTQMVSK.G
				1	797.57	1593.13	1592.77	0.36	0	115	1	U	K.MVGVPAALDMMLTGR.S + 2 Oxidation (M)
				1	797.59	1593.16	1592.79	0.37	0	112	1	U	R.FGGGNPELLTQMVSK.G + Oxidation (M)
					814.10	1626.19	1625.85	0.33	0	91	1	U	R.TIEYLEEVAITFAK.G
				1	816.07	1630.13	1629.83	0.30	0	107	1	U	K.TLQEVTQLSQEAQR.I
				1	544.52	1630.55	1629.83	0.72	0	83	1	U	K.TLQEVTQLSQEAQR.I
				1	840.63	1679.26	1678.85	0.41	0	109	1	U	K.ADMVIEAVFEDLSLK.H
					848.58	1695.15	1694.84	0.31	0	90	1	U	K.ADMVIEAVFEDLSLK.H + Oxidation (M)
					566.14	1695.41	1694.84	0.57	0	48	1	U	K.ADMVIEAVFEDLSLK.H + Oxidation (M)
				1	922.05	1842.085	1841.81	0.28	0	120	1	U	K.TGIEQGSDAGYLCESQK.F + Carbamidomethyl (C)
				1	1004.18	2006.35	2006.11	0.24	0	131	1	U	K.TVLGTPEVLLGALPGAGGTQR.L
					669.93	2006.77	2006.12	0.66	0	48	1	U	K.TVLGTPEVLLGALPGAGGTQR.L

	1	674.64	2020.90	2020.11	0.79	0	93	1	U	K.HLAILGAGLMGAGIAQVSVDK.G
	1	679.78	2036.31	2036.11	0.20	0	91	1	U	K.HLAILGAGLMGAGIAQVSVDK.G + Oxidation (M)
	1	1031.60	2061.19	2060.97	0.22	0	109	1	U	R.DSIFSNLTGQLDYQGFEK.A
		759.15	2274.42	2273.21	1.21	0	61	1	U	K.MGLVDQLVEPLGPGLKPPEER.T
	1	807.60	2419.77	2419.11	0.66	0	50	1	U	K.ELHSEFSEVMNEIWASDQIR.S
		812.84	2435.51	2435.11	0.40	0	41	1	U	K.ELHSEFSEVMNEIWASDQIR.S + Oxidation (M)
	1	903.15	2706.43	2706.38	0.04	0	48	1	U	K.STKPIVAAINGSCLGGGLEVAISCQYR.I
		922.12	2763.34	2763.27	0.07	1	41	1	U	K.TGIEQGSDAGYLCESQKFGELVMTK.E + Carbamidomethyl (C); Oxidation (M)
	1	946.29	2835.85	2835.49	0.36	1	120	1	U	K.KLDSLTTSFGFPVGAATLVDEVGVDVAK.H

SQRD_HUMAN Sulfide:quinone oxidoreductase, mitochondrial

Score	Mass	Matched ions	Peptide sequences	Dupes	m/z obs	M exp	M calc	Delta	Miss	Pept score	Rank	U	Peptide
705	49929	28	10										
				1	549.27	1096.53	1096.58	-0.05	0	56	1	U	R.VILAEFDYK.A
				3	655.32	1308.62	1307.62	1.00	0	60	1	U	K.IMYLSEAYFR.K + Oxidation (M)
				9	672.57	1343.13	1342.72	0.41	0	86	1	U	K.TAAAVAAQSGILDR.T
				2	724.65	1447.29	1447.73	-0.44	0	76	1	U	K.YADALQEIIQER.N
				2	755.05	1508.08	1507.72	0.36	0	71	1	U	K.AEPLETFPFDQSK.E
					789.28	1576.54	1575.83	0.71	1	60	1	U	K.KYADALQEIIQER.N
					883.64	1765.26	1764.78	0.48	0	69	1	U	K.YDGYTSCPLVTGYNR.V + Carbamidomethyl (C)
					947.74	1893.47	1892.87	0.59	1	66	1	U	K.KYDGYTSCPLVTGYNR.V + Carbamidomethyl (C)
					655.32	1962.93	1962.93	0.00	1	42	1	U	K.CAGAPQKIMYLSEAYFR.K + Oxidation (M)
				1	1009.68	2017.34	2017.01	0.34	0	125	1	U	R.NHYEVLVLGGGSGGITMAAR.M + Oxidation (M)

ATPA_HUMAN ATP synthase subunit α, mitochondrial														
**Score**	**Mass**	**Matched ions**	**Peptide sequences**											
531	59714	16	7	**Dupes**	**m/z obs**	**M exp**	**M calc**	**Delta**	**Miss**	**Pept score**	**Rank**	**U**	**Peptide**
					395.45	788.89	788.41	0.47	0	57	**1**	U	R.VGSAAQTR.A
					500.42	998.82	999.57	-0.75	0	46	**1**	U	R.VLSIGDGIAR.V
				1	586.36	1170.71	1170.62	0.08	0	75	**1**	U	R.VVDALGNAIDGK.G
				1	644.54	1287.06	1286.69	0.37	0	67	**1**	U	K.HALIIYDDLSK.Q
					659.21	1316.41	1315.73	0.68	0	89	**1**	U	K.TSIAIDTIINQK.R
				2	788.64	1575.27	1574.78	0.49	0	120	**1**	U	R.ILGADTSVDLEETG R.V
				5	812.80	1623.59	1623.88	-0.29	0	87	**1**	U	R.TGAIVDVPVGEELL GR.V

K6PP_HUMAN 6-phosphofructokinase, platelet type														
**Score**	**Mass**	**Matched ions**	**Peptide sequences**											
922	85542	47	15	**Dupes**	**m/z obs**	**M exp**	**M calc**	**Delta**	**Miss**	**Pept score**	**Rank**	**U**	**Peptide**
					427.91	853.81	853.44	0.37	0	41	1	U	R.MGIYVGAK.V + Oxidation (M)
					463.22	924.43	924.49	-0.07	0	43	1	U	K.LPDDQIPK.T
				4	511.87	1021.72	1021.60	0.11	0	69	1	U	R.VTILGHVQR.G
					529.55	1057.09	1057.56	-0.46	0	45	1	U	K.FLEHLSGAGK.A
					1058.50	1057.50	1057.56	-0.06	0	61	1	U	K.FLEHLSGAGK.A
					530.63	1059.25	1059.51	-0.26	1	48	1	U	K.KQTDFEHR.I
				1	534.35	1066.68	1066.55	0.13	0	50	1	U	R.TFVLEVMGR.H + Oxidation (M)
				1	557.87	1113.72	1113.55	0.17	0	50	1	U	R.SFAGNLNTYK.R
					396.29	1185.86	1185.65	0.21	1	41	1	U	R.KFLEHLSGAGK.A
				1	629.57	1257.12	1256.71	0.40	0	44	1	U	R.NVIFQPVAELK.K
				5	635.47	1268.93	1268.61	0.32	0	59	1	U	K.YLEEIATQMR.T + Oxidation (M)
				4	697.38	1392.74	1392.72	0.01	0	91	1	U	K.ELVVTQLGYDTR.V
				7	706.52	1411.02	1411.69	-0.67	0	72	1	U	R.DLQSNVEHLTEK. M

	1	732.51	1463.00	1462.68	0.32	0	83	1	U	R.MLAIYDGFDGFAK.G + Oxidation (M)
	1	601.48	1801.41	1802.89	-1.48	0	64	1	U	K.AIGVLTSGGDAQGMNAAVR.A + Oxidation (M)
	3	902.63	1803.25	1802.89	0.36	0	102	1	U	K.AIGVLTSGGDAQGMNAAVR.A + Oxidation (M)
	2	914.21	1826.40	1825.85	0.54	0	80	1	U	K.NVLGHMQQGGAPSPFDR.N + Oxidation (M)
